# A Non-invasive 24 Hours Stabilization of Duodenal Ulcer Perforation by a Combination Regimen

**DOI:** 10.7759/cureus.908

**Published:** 2016-12-01

**Authors:** Ahsan Zil-E-Ali, Muhammad Bin Shafique, Salman Assad, Hammad Ali, Usman Ghani

**Affiliations:** 1 Surgery, Fatima Memorial Hospital; 2 Department of General Surgery, Postgraduate Trainee, Ghurki Trust Hospital, Lahore Medical & Dental College; 3 Department of Medicine, Shifa Tameer-e-Millat University, Islamabad, Pakistan; 4 Biochemistry, Fatima Memorial Hospital; 5 Department of Medicine, Shifa International Hospital, Islamabad, Pakistan

**Keywords:** perforated ulcer, duodenal ulcer, non-invasive management

## Abstract

Surgical repair of perforated gastroduodenal ulcer has been extensively practiced in emergency clinical situations. Non-invasive conservation treatment is regaining the attention towards management of such ulcers. We report the case of a 50-year-old male smoker who presented in the emergency unit with acute generalized abdominal pain and guarding in the epigastric and right upper quadrant region. He is a known regular user of over-the-counter nonsteroidal anti-inflammatory drugs (NSAIDS) for more than 10 years for his osteoarthiritis and myalgias. A differential diagnosis of gastritis and duodenal perforation was made owing to the symptoms and long usage of NSAIDs. He was managed with an intravenous proton pump inhibitor and intravenous antibiotics. This therapy lead to stabilization of the clinical symptoms as well as laboratory and imaging studies.

## Introduction

Perforation of gastroduodenal ulcer complicates about two to five percent of the cases and carries a mortality rate up to 10%. Surgical repair with or without omental patch has been widely adapted as a therapeutic approach in perforated ulcers. In recent years, a conservation treatment approach to utilize a non-invasive and effective management of perforated duodenal ulcer has gained attention [1]. A conservative management consisting of effective gastric decompression, fluid resuscitation, and administration of anti-secretory agents along with broad spectrum antibiotics is a reasonable approach for selective patients with perforated gastroduodenal ulcers [2]. In this case report, we describe a patient with perforated duodenal ulcer who was treated conservatively without the development of any complications. Informed consent was obtained from the patient for this study.

## Case presentation

A 50-year-old male smoker presented in the emergency unit with acute generalized abdominal pain and guarding in the epigastric and right upper quadrant region. The patient complained of abdominal pain for the last 12 hours with two episodes of vomiting in the last five hours and complete constipation for two days. The patient is a known regular user of over-the-counter nonsteroidal anti-inflammatory drugs (NSAIDS) for more than 10 years for his osteoarthritis and myalgias.

The patient had a distended centrally inverted abdomen with thoraco-abdominal respiratory movements. A dull percussion note was present at the flanks with decreased bowel sounds. No visceromegaly was noted on physical examination. The patient was febrile with a temperature of 100℉ though the rest of his vitals were stable with a heart rate of 87 per minute, respiratory rate of 17 per minute and blood pressure of 130/90 mmHg. A pre-rectal examination showed a collapsed rectum, normal prostate palpation and tenderness on deep bimanual palpation.

After a brief history and physical examination in the emergency room, the patient was admitted and a thorough workup panel was requested. The complete blood panel showed neutrophilic leucocytosis, though renal function tests, liver function tests, urine complete analysis, serum electrolytes and erythrocyte sedimentation rates were all within normal range. Further workups for hepatitis B antigen and antibody for hepatitis C showed no viral antigenicity. Serology for helicobacter pylori was also negative.

**Figure 1:**
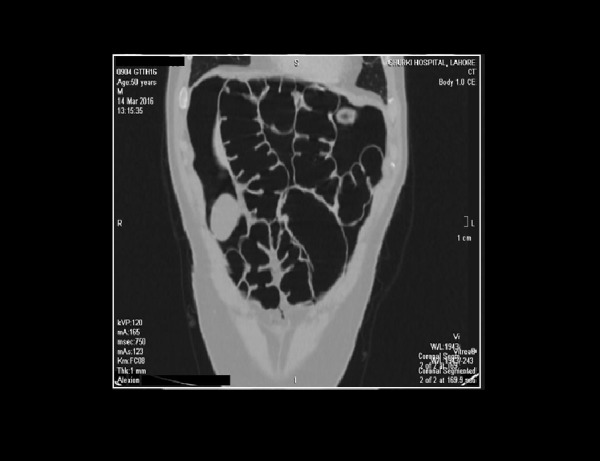
A coronal section of the CT abdomen showing pneumo-peritoneum along with pneumatosis intestinalis and thick reactive intestine walls. The radiologic presentation assures the presence of air in the gut, which can be due to a perforation.

**Figure 2:**
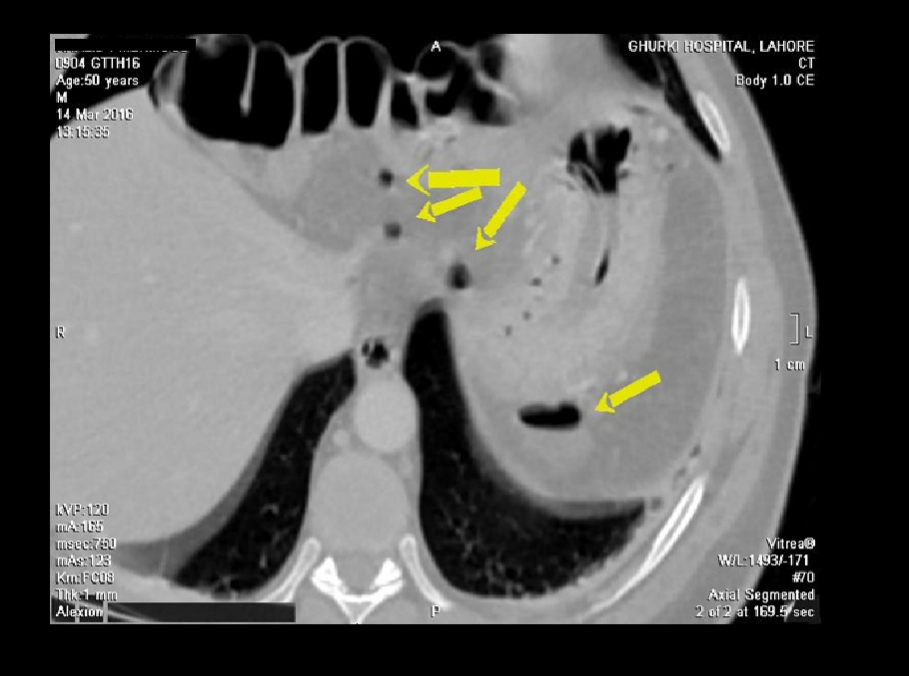
CT scan of the abdomen (axial segment): pneumo-peritoneum and a few air pockets (yellow arrows) are appreciated around the stomach, which may be most likely a result of gastric perforation.

**Figure 3:**
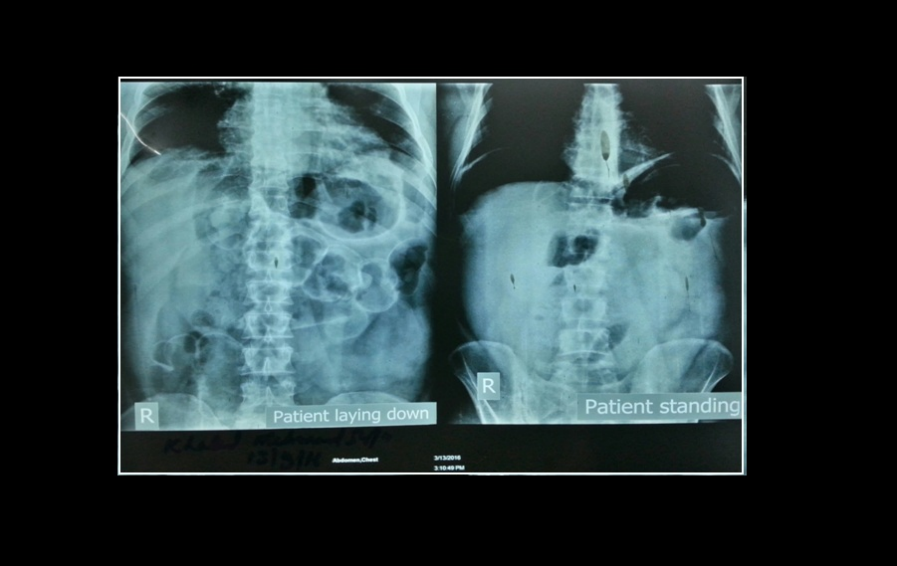
X-ray abdomen of the patient in supine (L) and standing (R) showing double wall appearance of the intestines (Rigler’s sign) with a clear liver edge and air under the diaphragm (‘Football’ sign). In the standing anteroposterior view, a bubble-like low density patch can be seen in the duodenal region showing a perforated site.

**Figure 4:**
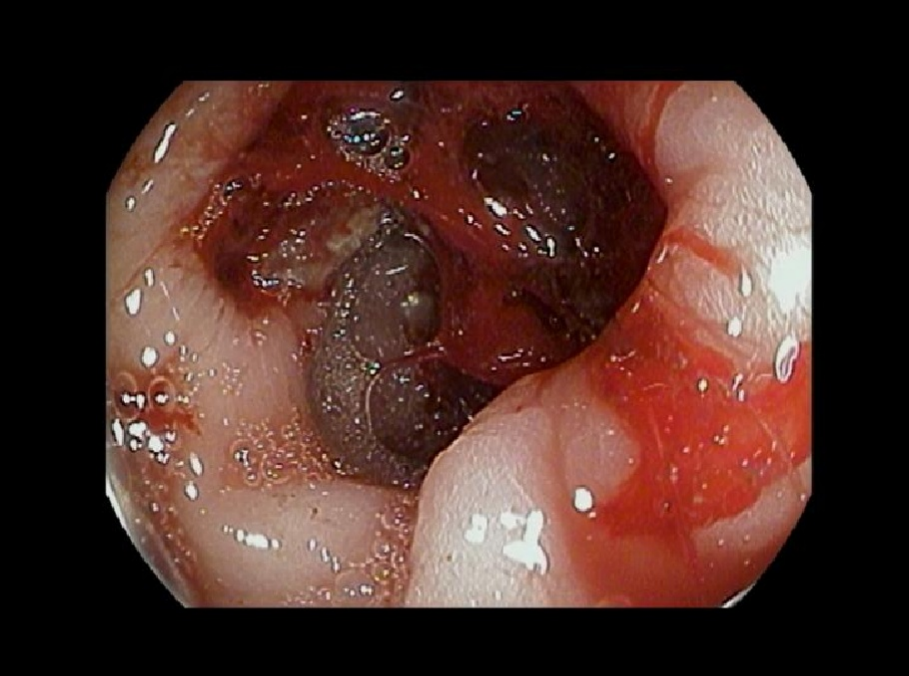
The endoscopic picture at the second part of the duodenum showing blood oozing from the perforated site, although the omentum covered the site of perforation. The perforation is in the posterior wall of the duodenum and is most likely at the junction of the second and third part.

A differential diagnosis of gastritis and duodenal perforation was made owing to the symptoms and long usage of NSAIDs. Pancreatitis, biliary pathologies and bacteremia were considered second options in finalizing the diagnosis. The patient was sent for a radiological consult where his radiographs, abdominal ultrasound, and computerized tomography were done (Figures 1-4).

After making the final diagnosis, the surgical team decided to treat the patient with a unique and non-invasive approach. To start the management, a nasogastric tube was passed and all the gastric contents were removed. This step requires special expertise as an improper removal of gastric contents will hamper this method. An intravenous administration of a proton pump inhibitor, Risek™ (omeprazole) 40 mg over 24 hours and H2 blocker, Zantac™ (ranitidine) 150 g over 24 hours were initiated. Along with these anti-gastric acid therapies, an intravenous injectable antibiotic Tanzo™ (tazobactam sodium) was given every eight hours. The patient was closely monitored for addressing any signs and symptoms. A surgical team was prepared as a backup for any invasive procedure in case of failure of this conservative management. During the course of 24 hours, the patient’s symptoms were gradually alleviated and a drastic drop in white blood cells was also observed. This approach brought acceptable results.

The abdominal girth was measured and regular palpations were done to check for any reoccurrence of the symptoms. The patient passed flatus on the third day and passed stool on the fourth day of admission. The patient graded 0/10 on the pain scale on the fifth day. Ultrasound was repeated and no signs of free air or fluid were seen. The patient was advised to consume semisolid food items and was discharged on the ninth day of admission. An anti-ulcer therapy with a proton pump inhibitor, oral tablet Risek™ (omeprazole) was prescribed for the next six weeks.

## Discussion

In 1843, Edward Crisp described the process of self-healing ulcers by adhesion formation that prevented leakage of gastric contents into the peritoneum. Later, in 1935, Wangensteen reported a case series of seven patients who recovered from perforated ulcers by self-healing. In 1946, Herman Taylor first confirmed this process when he reported 28 patients with perforated ulcers who were treated conservatively by nasogastric aspiration, intravenous (IV) fluids and serial abdominal X-rays (now known as Taylor’s method) [1]. Taylor reported successful outcomes with his conservative approach, with a mortality of 10%.

The efficiency of Taylor’s method was established by Dascalescu C, et al., who conducted a retrospective study on prospectively collected data consisting of 64 patients with perforated gastroduodenal ulcers who were treated non-operatively. The diagnosis of perforated ulcer was made by clinical presentation, as well as radiological findings of pneumoperitonium on erect chest X-ray and abdominal ultrasound. The conservation treatment approach consisted of a nasogastric tube, intravenous fluids, broad spectrum antibiotics and anti-secretory drugs. This study reported a success rate of 89% with the use of Taylor’s method in managing perforated ulcers. The most common complication encountered was intra-abdominal abscess, and no mortalities were reported [2]. The diagnosis of perforation in the patient described in our case was done by erect chest X-ray, CT abdomen and confirmed with endoscopy. The patient successfully underwent conservative management with no complications. Patients who develop abscesses as a complication can be treated with antibiotics and drainage.

The decision of operative versus conservation therapy depends on the patient's hemodynamic status and overall condition. Donovan, et al. published a selective treatment protocol for patients with duodenal ulcer perforation in 152 patients [3]. A non-operative approach was used in those patients with acute perforated ulcers with spontaneous sealing of perforation, or a chronic ulcer in a patient with very poor surgical risk. The presence or absence of the seal was confirmed by performing a gastroduodenogram. The criteria for spontanous sealing was absence of intraperitonal contrast spilling, which is used to select patients who are likely to respond well to conservative therapy. An extraduodenal leak of contrast is an indication for surgical management [3]. In our patient, we successfully stabilized his condition with conservation therapy, without the need to perform a contrast study for confirming the sealing of perforation.

Crofts TJ, et al. conducted a prospective randomized trial comparing the outcomes of conservative treatment versus surgical treatment in 83 patients with perforated gastric ulcer. In this study, 40/83 patients were randomly treated conservatively with Taylor’s method and 43/83 underwent surgical repair. The study reported similar mortality and morbidity rates in both the groups (five percent each, and 40% vs 50%). The study concluded that patients with perforated peptic ulcer may be observed in the initial 24 hours and managed non-operatively [4]. The exception to this was patients older than 70 years of age, which was a factor associated with higher risk of surgical intervention. Other factors such as shock (hypotension) and comorbidities have also been described as factors contributing to poor response to conservative approach, as well as associated with higher mortality [5-6].

Non-operative treatment approach for perforated ulcers has remained controversial despite being a safe and efficacious option. However, it is important to adhere to protocols while managing patients with perforated ulcers. A retrospective study by Marshall C, et al. reported poor adherence to protocol guidelines. These guidelines included endoscopic diagnosis, the use of antibiotic regimen and the importace of follow-up endoscopy for detection of potential peptic ulcers [7]. Our patient underwent diagnostic endoscopy to establish an accurate diagnosis of perforated ulcer before being considered for conservation management. When a gastroduodenogram shows self-healing of perforation, patients can be safely managed with non-operative measures. After a proven sealing of perforation site, non-surgical management was considered safe, with a three percent complication of abscess formation and less than two percent rate of re-leak [3].

More than half of the patients with perforated gastroduodenal ulcer will seal spontaneously [1, 3-4]. The initial management of perforated duodenal ulcer involves stabilizing the patient with nasogastric decompression, volume repletion, administration of antibiotics and proton pump inhibitors (PPI). The antibiotic regimen should be broad spectrum, covering gram-negative rods, anerobes, and oral flora. A reasonable choice of antibiotic would be ampicillin-sulbactam, ticarcillin-clavulanic acid or piperacillin-tazobactam. Alternate regimens include monotherapy with carbapenems, or a combination therapy with third generation cephalosporin plus metronidazole for anerobic coverage. A serious concern with the use of conservation management is the risk for incorrect diagnosis. Misdiagnosis can result in high mortality rates, as well as prolonged hospital stays [8].

This is apparent with the worsening of a patient’s condition and peritonitis. Our patient in this case was stabilized with conservative management, without any signs of clinical deterioration. It is therefore suggested, as described in this case also, to perform repeated physical examinations to reassess the patient’s condition for signs of deterioration.

## Conclusions

A conservative approach with Taylor’s method is a reliable approach in selective patients with perforated gastroduodenal ulcers. Careful observation for the first 24 hours and early administration of Taylor’s method is a reasonable approach for patients with perforated duodenal ulcer. Any signs of clinical deterioriation, shock or comorbid illnesses warrant transfer to surgical care. Strict guidelines and protocols are recommended before initiating a conservative approach.
